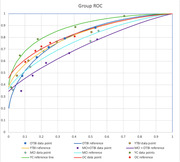# Behavioral estimates of recognition memory in Veterans with mild TBI at risk of Neurodegeneration

**DOI:** 10.1002/alz.090339

**Published:** 2025-01-09

**Authors:** Kristina Morreale, Meltem Karaca, Myna Chadalavada, Emily Waskow, Brenna Hagan, Renée DeCaro, Natalie Anderson, Andrew E. Budson, Katherine W. Turk

**Affiliations:** ^1^ VA Boston Healthcare System, Jamaica Plain, MA USA; ^2^ Boston University School of Medicine, Boston, MA USA; ^3^ Boston VA, Boston, MA USA; ^4^ Boston University Chobanian & Avedisian School of Medicine, Boston, MA USA

## Abstract

**Background:**

Loss of recognition memory has been hypothesized to be a potential behavioral marker of neurodegenerative disease given decreases in both recollection and familiarity reported in both MCI (Mild Cognitive Impairment) and AD (Alzheimer’s Disease). Stage III CTE (Chronic Traumatic Encephalopathy) involves p‐tau deposition in the medial temporal lobe, impacting memory. Decreases of both familiarity and recollection are promising potential markers of CTE related neurodegeneration following TBI (Traumatic Brain Injury). Decreases in recollection may act as an early marker of neurodegeneration following TBI in CTE, whereas familiarity may act as a later stage indicator of neurodegeneration.

**Method:**

We collected behavioral data including memory measures, neuropsychological measures, head injury exposure, and standardized questionnaires. Veteran participants were recruited from VA Boston Healthcare System, N = 45.

**Result:**

We analyzed initial behavioral data with specific focus on group levels of memory using receiver operator characteristics curves (see Figure 1). Pearson correlations were performed on groups with TBIs across their lifetimes to assess the linear relationship between a.) number of TBIs and familiarity r (43) = ‐.107, p < .001, b.) years since last TBI and recollection r (43) = ‐.107, p < .001, and c.) years since last TBI and familiarity r (43) = ‐.139, p < .001. A one‐way ANOVA was preformed to examine if groups differed in measures of recollection, familiarity, number of TBIs and years since last TBI. MCI + OTBI a.) group showed the worst recollection compared to young adults with TBI F(2,41) = 3.33, p = .046, b.) highest number of TBIs than older adults with TBI F( 2,41) = 3.65, p = .036, and c.) have more years elapsed as compared to all head injury groups F( 2,41) = 8.12, p = .001.

**Conclusion:**

Our results reveal worsened memory in those with TBIs. Among all Veterans with TBI, increased number of TBIs were associated with worse familiarity performance. Participants with greater years since last TBI had worse familiarity and recollection performance. Between groups, the MCI participants with TBI (MCI+OTBI) exhibited the worst familiarity performance, highest number of TBIs, and longest time since head injury.